# Appraisal of anti-arthritic and nephroprotective potential of *Cuscuta reflexa*

**DOI:** 10.1080/13880209.2017.1280513

**Published:** 2017-01-19

**Authors:** Alamgeer, Samia Gul Niazi, Ambreen Malik Uttra, Muhammad Naeem Qaiser, Haseeb Ahsan

**Affiliations:** Department of Pharmacology, Faculty of Pharmacy, University of Sargodha, Sargodha, Pakistan

**Keywords:** Turpentine oil, formaldehyde, denaturation, phytochemical

## Abstract

**Context:** *Cuscuta reflexa* Roxb. (Cuscutaceae) has been used traditionally for treating sore knees and kidney problems, but its efficacy has not been scientifically examined in treating arthritis and nephrotoxicity.

**Objective:** Present study determines antiarthritic and nephroprotective potential of the aqueous methanolic extract of *Cuscuta reflexa* (AMECR).

**Materials and methods:** Antiarthritic activity of *Cuscuta reflexa* in formaldehyde and turpentine oil-induced rat arthritis models was appraised at 200, 400 and 600 mg/kg doses for 10 days and 6 h period, respectively, and *in vitro* protein denaturation (bovine serum albumin, egg albumin) inhibition was studied at 25–800 μg/mL concentration. The nephroprotective effect involved gentamicin-induced nephrotoxicity in rats at 200, 400 and 600 mg/kg doses.

**Results:** Plant extract at 600 mg/kg significantly reduced paw oedema and joint swelling with maximal inhibition of 71.22% at the 6th hour for turpentine oil and 76.74% on 10th day for formaldehyde. Likewise, *in vitro* results corroborated significant concentration-dependent increase in percentage protection at 800 μg/mL against both bovine serum albumin (89.30%) and egg albumin (93.51%) denaturation. Similarly, 600 mg/kg dose showed maximum nephroprotection by reducing serum urea (41.400 ± 0.510 mg/dL), uric acid (0.740 ± 0.032 mg/dL), blood urea nitrogen (18.370 ± 0.328), creatinine (3.267 ± 0.076) and minimizing kidney weight gain (0.586 ± 0.005) and histopathological alterations on 8th day. Furthermore, phytochemical and HPLC analysis revealed the presence of important phytoconstituents.

**Discussion and conclusions:** These results suggest that AMECR provides protection against arthritis and nephrotoxicity that might be due to the existence of phytoconstituents, thus supporting folkloric claim.

## Introduction

Arthritis is a combination of two Greek words ‘Arthro’ means joint and ‘Itis’ means inflammation. So, persistent joint synovial fluid inflammation is termed as rheumatoid arthritis (RA) (Kalla et al. 2003). In RA, body’s immune system attacks body’s own tissues, hence it not only effects joints but also effects other parts of body such as skin, eyes, lungs and nerves (Efthimiou & Kukar 2010). Hallmark of RA is persistent symmetric polyarthritis resulting in muscle aches and pain. Anti-citrullinated protein antibodies (ACPA) produced by plasma cells during the preclinical phase of RA, stimulate osteoclast differentiation while, synovitis at the onset of disease leads to the production of cytokines, thus ensuing in bone erosion (Boldt et al. 2012). All current therapies for RA have certain shortcomings. So, due to easy and continuous availability, better compatibility (Kamboj 2000), cost effectiveness, less potential of toxicity and side effects, higher safety, and improved efficacy of herbal medicines (Khanna et al. 1986), they are being preferred over conventional drugs nowadays. Nephrotoxicity, on the other hand, can be defined as the development of structural and functional kidney damage as a result of exposure to external stimuli such as, any drug or environmental chemicals. Renal failure is characterized by decrease in GFR, azotemia, changes in urine volume and biochemical homeostatic processes (El-Tantawy et al. 2013). Pakistan is gifted with rich source of medicinal plants. *Cuscuta reflexa* Roxb. (Cuscutaceae) is obligate twining climber holoparasite (Bleischwitz et al. 2010). In Urdu it is named as Akashbel. *Cuscuta reflexa* has been used traditionally for the treatment of sore knees and kidney problems (Vijikumar et al. 2011; Udavant et al. 2012). The present study was designed for the scientific confirmation of the traditional use of *Cuscuta reflexa* in arthritis and nephrotoxicity by local populace using various *in vitro* and *in vivo* models.

## Materials and methods

### Chemicals

Formalin (VWR, International Ltd, Poole, England), turpentine oil (UNI-CHEM, Germany), diclofenac sodium (Sigma-Aldrich, St. Louis, MO), aspirin (Sigma-Aldrich Laborchemikalien GmbH, Seelze), bovine serum albumin (Sigma-Aldrich, St. Louis, MO), egg albumin from fresh hen’s egg, gentamicin sulphate 40 mg/mL (Novartis Pharmaceutical Ltd.).

### Plant material and extraction

*Cuscuta reflexa* whose stems were growing on the plant of *Ziziphus* was collected in October 2014 from the local region of Faisalabad district. The plant was identified and authenticated by Dr. Mansoor Ahmed, Department of taxonomy, University of Agriculture, Faisalabad. Voucher specimen of this plant was deposited under voucher number 225-1 in the herbarium of Faculty of Pharmacy, University of Sargodha, for future reference. The plant was collected from the aerial parts of *Ziziphus*, so it was separated and properly washed with water. After shade-drying, plant was chopped and grinded in mortar and pestle, and finally grinded in a electrical grinder to a fine powder. The grounded plant material was soaked in a water:methanol mixture (30:70) at room temperature. After three days of occasional shaking, whole material was filtered through Whattman-No.1 filter paper and the filtrate was evaporated under reduced pressure using rotary evaporator. The crude extract (AMECR) was then air-dried to obtain a solid mass (Alamgeer et al. 2013).

### Experimental animals

Sprague–Dawley rats of either sex, weighing 150–200 g were used for *in vivo* study. The animals were housed in stainless steel cages under controlled room temperature, allowed free access to diet and water and received human care according to the guidelines of National Institute of Health (NRC 1996). The use of animals in the study followed principles from the Declaration of Helsinki. The use of animals in the study was approved by Institutional Animal Ethics Committee Faculty of Pharmacy, University of Sargodha (Approval No. 20A26 IEC UOS).

### Solubility analysis of the aqueous methanolic extract of *Cuscuta reflexa*

Solubility analysis of AMECR was conducted in distilled water, methanol, ethanol, ethanol:water, *n-*hexane and 5% DMSO. An equal amount of extract (up to 0.2 g) was weighed on digital weighing balance and taken in different Eppendorf tubes. Each solvent (1 mL) was poured into Eppendorf tubes and shaken vigorously on a vortex mixture for 2–4 min. Then, the solution was allowed to settle down and solubility was determined by observing the presence of any sediment material.

### Evaluation of antiarthritic effect of *Cuscuta reflexa* on formaldehyde induced arthritis

All the animals were kept under standard conditions in animal house in neat and clean cages for 7 days and given standard diet. Briefly, after 7 days the animals were divided into five groups (*n* = 5) (Singh et al. 2010). Group I: Formaldehyde (2%) 0.1 mL at 1st and 3rd day plus 3 mL/kg distilled water. Group II: Formaldehyde (2%) 0.1 mL for 1st and 3rd day plus standard aspirin (100 mg/kg). Group III, IV, V: Formaldehyde (2%) 0.1 mL for 1st and 3rd day plus AMECR (200, 400, 600 mg/kg/d, respectively). All the groups received formaldehyde after half an hour of oral administration of extract or vehicle into the sub-plantar surface of right hind paw. Arthritis was determined by measuring mean increase in the paw diameter of animals for a period of 10 days using digital vernier caliper. Percentage inhibition of paw oedema was calculated using the following formula (Kumar et al. 2013).

$$\mathrm{Percentage}\;\mathrm{inhibition}\;=\;\frac{\mathrm{VC}-\mathrm{VT}}{\mathrm{VC}}\;\times \;100$$

VC: paw oedema of the control group, VT: paw oedema of the test group

### Evaluation of antiarthritic effect of *Cuscuta reflexa* on turpentine oil-induced arthritis

Animals were fasted over night before the experiment. They were divide into five groups (*n* = 5). Group I: turpentine oil (0.02 mL) plus 3 mL/kg distilled water. Group II: turpentine oil (0.02 mL) plus aspirin (100 mg/kg). Group III, IV, V: turpentine oil (0.02 mL) plus AMECR (200, 400, 600 mg/kg, respectively). Joint oedema was developed by injecting turpentine oil into the synovial cavity of right knee joint, 30 min after oral administration of drug. Joint diameter was recorded at hourly intervals for 6 h with digital vernier caliper (Kumar et al. 2013). Percentage inhibition of knee joint oedema was determined using the following formula:

$$\mathrm{Percentage}\;\mathrm{inhibition}\;=\;\frac{\mathrm{VC}\;-\;\mathrm{VT}}{\mathrm{VC}}\;\times \;100$$

VC: paw oedema of control group, VT: paw oedema of test group

### Evaluation of antiarthritic effect of *Cuscuta reflexa* on inhibition of protein denaturation (fresh hen’s egg albumin)

Reaction mixture (5 mL) was prepared having 0.2 mL of fresh eggs albumin plus 2.8 mL of phosphate buffer saline (pH 6.3) plus 2 mL of varying concentrations (25, 50, 100, 200, 400 and 800 μg/mL) of AMECR. Similar volume of double-distilled water served as control. Reaction mixtures were incubated at 37 ± 2 °C in a BOD incubator for 15 min and heated at specified temperature of 70 °C for 5 min. After cooling, absorbance was measured at 660 nm using UV visible spectrophotometer. Diclofenac sodium at similar concentrations was used as reference drug (Chandra et al. 2012). The percentage inhibition of protein denaturation was calculated using the following formula:

% inhibition=(Abs control-Abs sample)×100/Abs control

### Evaluation of antiarthritic effect of *Cuscuta reflexa* on inhibition of protein denaturation (bovine serum albumin)

Test solution (0.5 mL) consisted of 0.05 mL of AMECR at various concentrations (25–800 μg/mL) and 0.45 mL of BSA (5% aqueous solution). Test control solution (0.5 mL) consisted of 0.05 mL of distilled water and 0.45 mL of BSA (5% aqueous solution). Product control solution consisted of 0.05 mL of AMECR at different concentrations (25–800 μg/mL) and 0.45 mL of distilled water. Standard solution (0.5 mL) consisted of 0.05 mL aspirin (25–800 μg/mL) plus 0.45 mL of BSA (5% aqueous solution). Solutions were adjusted to pH 6.3 by 1 N HCl and incubated at specific temperature (37 °C) for 20 min and temperature was increased progressively up to 57 °C for 3 min. Solutions were cooled for 25 min and then 2.5 mL of phosphate buffer was added to all the solutions. After buffering, resultant solution absorbance was measured using UV visible spectrophotometer at 416 nm (Sheelarani et al. 2014). The percentage inhibition of protein denaturation was determined using the following formula:

$$\mathrm{Percentage}\;\mathrm{inhibition}:\;100\;-\;\frac{\mathrm{Absorbance}\;\mathrm{of}\;\mathrm{test}\;\mathrm{soln}.\;-\;\mathrm{Absorbance}\;\mathrm{of}\;\mathrm{product}\;\mathrm{control}}{\mathrm{Absorbance}\;\mathrm{of}\;\mathrm{Test}\;\mathrm{Control}\;\mathrm{soln}.}\;\times \;100$$

### Evaluation of nephroprotective effect of *Cuscuta reflexa* against gentamicin induced nephrotoxicity

Rats were divided into 5 groups (*n* = 5). Group I: Normal control (normal saline). Group II: Toxic control (Gentamicin 100 mg/kg). Group III, IV, V: Gentamicin (100 mg/kg) plus AMECR (200, 400, 600 mg/kg/d, respectively) (Lakshmi & Sudhakar 2010).

On the 8th day of drug administration, rats were killed with chloroform and blood was collected by cardiac puncture. It was then centrifuged at 370 rpm for 10 min and then refrigerated at −20 °C for biochemical analysis. The samples were analyzed for blood urea nitrogen, serum urea, creatinine and uric acid using standard kits. The kidneys were removed from each rat and their weight was recorded. Moreover, pieces of kidney of each rat were fixed immediately in 10% neutral formalin for 24 h and sections were evaluated for pathological symptoms of nephrotoxicity (Preethi & Kuttan 2009).

### Preliminary phytochemical analysis of aqueous methanolic extract of *Cuscuta reflexa*

For preliminary phytochemical analysis, standard procedures were performed. To examine the presence of flavonoids and saponins, method of Roopashree et al. (2008) was followed. Similarly, method of Tadesse et al. (2012) was used to confirm the presence of alkaloids, terpenoids and anthraquinones. While, method followed by Abbas et al. (2012) was used to detect the presence of tannins.

### HPLC analysis of aqueous methanolic extract of *Cuscuta reflexa*

To 50 mg of AMECR, 16 mL of deionized distilled water and 24 mL of methanol was added. The solution was shaken for 5 min and 10 mL of 6 M HCl was added and kept in an oven at 90 °C for 2 h. It was then filtered through 0.2–0.4 μ filter paper. This sample was then run in HPLC for determining flavonoid and phenolic compound in the plant extract using UV visible detector. Shim-pack CLC-ODS (C-18) column of dimensions 25 cm ×4.6 mm was used. Mobile phase consisted of 2 solutions, i.e., Solution A = (H_2_O:AA (acetic acid) – 94:6, of pH = 2.27 and Solution B = CAN (Acetonitrile 100%). The mobile phase was moved through column at the rate of 1 mL/min for 10 min. The peak was observed at 280 nm using UV visible detector (Sheth & De 2012).

### Statistical analysis

The results were expressed as mean ± SEM and data were analyzed statistically using two-way ANOVA followed by the Bonferroni post-test using Graph Pad Prism 5.0. *p* < 0.05 was considered significant.

## Results

### Solubility analysis of *Cuscuta reflexa*

The solubility analysis was performed to determine particular solvent in which the extract is soluble. The extract of *Cuscuta reflexa* was highly soluble in methanol and soluble in 5% DMSO while, sparingly soluble in distilled water and ethanol but insoluble in ethanol:water and *n*-hexane.

### Effect of *Cuscuta reflexa* against formaldehyde induced arthritis

The results of this experiment (Table 1) depict that AMECR on 10th day at 600 mg/kg (maximum dose) showed significant (*p* < 0.001) reduction in paw oedema, i.e., 76.74% as compared to arthritic control group and these results were almost comparable with standard drug aspirin (77.32%). However, 400 and 200 mg/kg doses exhibited 73.20% and 71.10% reduction in paw diameter, respectively.

**Table 1.** Effect of AMECR on formaldehyde induced arthritis in Sprague–Dawley rats.

	Change in paw diameter (mm)				
Treatment groups	Day 2	Day 4	Day 6	Day 8	Day 10
Arthritic Control (3 mL/kg)	8.480 ± 0.046	10.417 ± 0.220	13.767 ± 0.377	16.317 ± 0.296	18.167 ± 0.300
Standard aspirin (100 mL/kg)	5.050 ± 0.050*** (40.44%)	5.667 ± 0.060*** (45.59%)	4.800 ± 0.153** (65.13%)	4.267 ± 0.093*** (73.84%)	4.120 ± 0.040*** (77.32%)
AMECR 200 mg/kg	7.167 ± 0.101*** (15.48%)	6.767 ± 0.101*** (35.03%)	5.800 ± 0.760*** (57.87%)	5.400 ± 0.870*** (66.90%)	5.250 ± 0.290*** (71.10%)
AMECR 400 mg/kg	6.550 ± 0.058*** (22.75%)	6.417 ± 0.120*** (38.39%)	5.433 ± 0.093*** (60.53%)	5.050 ± 0.050*** (69.06%)	4.867 ± 0.044*** (73.20%)
AMECR 600 mg/kg	6.250 ± 0.058*** (26.29%)	6.050 ± 0.029*** (41.92%)	5.067 ± 0.44*** (63.19%)	4.690 ± 0.097*** (71.25%)	4.225 ± 0.025*** (76.74%)

Values in the parenthesis represent percentage inhibition of paw oedema. Results are expressed as mean ± SEM (*n* = 5), using two way ANOVA followed by the Bonferroni post-test.

*** *p* < 0.001 when compared to control.

### Effect of *Cuscuta reflexa* against turpentine oil induced arthritis

The results given in Table 2 express a highly significant (*p* < 0.001) reduction of joint cavity swelling by all treatment groups at the end of study period when compared to the arthritic control group. At 6th hour, AMECR at a dose of 600 mg/kg (highest dose) presented maximum suppression of knee joint oedema, i.e., 71.22%, while standard drug aspirin displayed 71% reduction in oedema. Moreover, 400 and 200 mg/kg demonstrated 68.01% and 66.57% reduction, respectively.

**Table 2.** Effect of AMECR on turpentine oil induced arthritis in Sprague–Dawley rats.

	Change in joint diameter (mm)					
Treatment groups	1st hour	2nd hour	3rd hour	4th hour	5th hour	6th hour
Arthritic control	8.893 ± 0.087	13.013 ± 0.373	16.433 ± 0.262	17.917 ± 0.203	19.000 ± 0.126	21.083 ± 0.484
Standard aspirin	7.207 ± 0.012*** (18.95%)	6.843 ± 0.047*** (47.41%)	7.367 ± 0.101*** (59.95%)	6.580 ± 0.012*** (63.27%)	6.320 ± 0.012*** (66.73%)	6.173 ± 0.018*** (71%)
AMECR 200 mg/kg	7.770 ± 0.067*** (12.62%)	7.747 ± 0.039*** (40.46%)	7.543 ± 0.015*** (54.09%)	7.367 ± 0.018*** (58.88%)	7.230 ± 0.026*** (61.94%)	7.047 ± 0.032*** (66.57%)
AMECR 400 mg/kg	7.430 ± 0.015*** (16.45%)	7.327 ± 0.09*** (43.69%)	7.243 ± 0.022*** (55.92%)	7.090 ± 0.017*** (60.42%)	6.913 ± 0.019*** (63.61%)	6.743 ± 0.043*** (68.01%)
AMECR 600 mg/kg	6.993 ± 0.060*** (21.36%)	6.667 ± 0.056*** (48.76%)	6.453 ± 0.019*** (60.73%)	6.243 ± 0.022*** (65.15%)	6.120 ± 0.015*** (67.70%)	6.067 ± 0.01*** (71.22%)

Values in the parenthesis represent percentage inhibition of joint oedema. Results are expressed as mean ± SEM (*n* = 5), using two-way ANOVA followed by the Bonferroni post-test.

*** *p* < 0.001 when compared to control.

### Effect of *Cuscuta reflexa* on protein denaturation using fresh hen’s egg albumin

**Figure 1.**
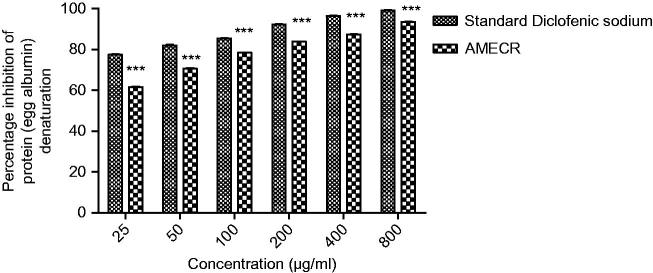
Effect of AMECR on percentage inhibition of protein (egg albumin) denaturation. All the values are expressed as mean ± SEM (*n* = 3), using two-way ANOVA followed by the Bonferroni post-test. ***(*p* < 0.001) vs standard group.

Figure 1 illustrates a concentration-dependent inhibition of protein denaturation by AMECR and standard diclofenac at a dose range of 25–800 μg/mL. Maximum inhibition was shown at 800 μg/mL in the case of both AMECR and diclofenac, i.e., 93.51 and 99.16%, respectively.

### Effect of *Cuscuta reflexa* on protein denaturation using bovine serum albumin

**Figure 2.**
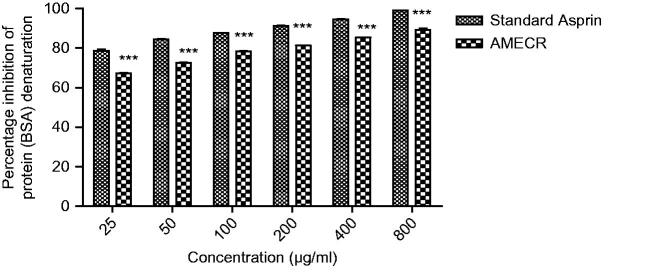
Effect of AMECR on percentage inhibition of protein (bovine serum albumin) denaturation. All the values are expressed as mean ± SEM (n = 3), using two-way ANOVA followed by the Bonferroni post-test. ***(*p* < 0.001) vs standard group.

AMECR at several concentrations (25–800 μg/mL) provided a considerable protection against the denaturation of BSA. The results summed up in Figure 2 explain that higher concentration of AMECR and reference drug aspirin showed maximum percentage inhibition of protein denaturation, i.e., 89.30 and 99.12%, respectively. However, lowest concentration also exhibited appreciable results.

### Effect of *Cuscuta reflexa* against gentamicin induced nephrotoxicity

Nephroprotective activity of AMECR was determined by analyzing biochemical, physical and histopathological parameters. AMECR expressed a highly significant nephroprotective effect when compared with toxic control group animals. Gentamicin treatment for 8 days ensued significant increase in the serum level of uric acid, urea, blood urea nitrogen and creatinine in control rats. Conversely, elevations in the level of these markers were significantly (*p* < 0.001) attenuated by AMECR pretreatments, indicating reduction in gentamicin-induced nephrotoxicity. Maximum protective effect was demonstrated at a dose of 600 mg/kg, followed by 400 and 200 mg/kg (Table 3). Furthermore, AMECR at a dose of 600 mg/kg preserved the kidney weight to near normal control group (Table 4).

**Table 3.** Biochemical parameters serum urea, serum creatinine, blood urea nitrogen and serum uric acid.

Biochemical Parameters (mg/dL)	Normal control	Toxic control	AMECR 200 mg/kg	AMECR 400 mg/kg	AMECR 600 mg/kg
Serum urea	43.400 ± 0.510***	61.800 ± 0.583***	48.400 ± 0.510***	44.600 ± 0.509***	41.400 ± 0.510**
Blood urea nitrogen	18.397 ± 0.088***	34.593 ± 0.547***	24.946 ± 0.659***	22.896 ± 0.257***	18.370 ± 0.328***
Serum creatinine	4.310 ± 0.073***	5.714 ± 0.126***	4.676 ± 0.068***	3.968 ± 0.034***	3.267 ± 0.076**
Serum uric acid	0.654 ± 0.021***	1.878 ± 0.048*	0.909 ± 0.013***	0.865 ± 0.017***	0.740 ± 0.032***

Values are expressed as ± SEM (*n* = 5), by two-way ANOVA and *p*<0.05 was considered as significant as compared to toxic control where

*** *p* < 0.001

** *p* < 0.01.

**Table 4.** Effect of *Cuscuta reflexa* on kidney weight of rats.

Group of Animals	Mean kidney weight (g)
Normal control	0.544 ± 0.011
Toxic control	0.702 ± 0.009
AMECR 200 mg/kg	0.624 ± 0.009
AMECR 400 mg/kg	0.606 ± 0.009
AMECR 600 mg/kg	0.586 ± 0.005

**Figure 3.**
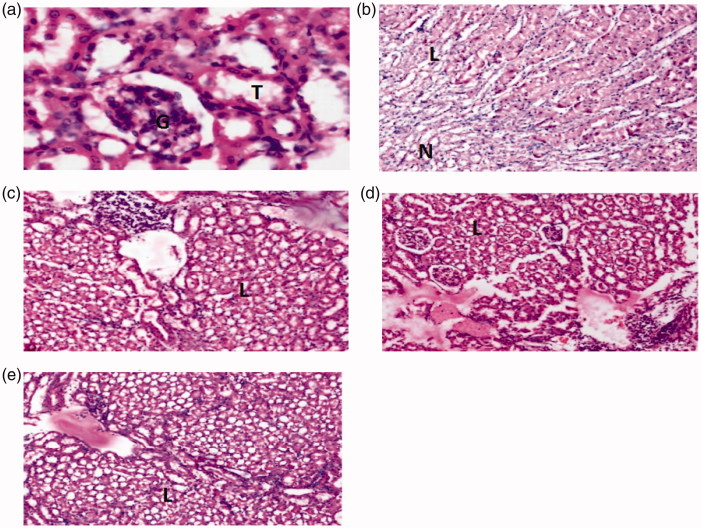
Histopathological analysis of the kidneys of Sprague–Dawley rats at the 8th day of treatment after gentamicin-induced nephrotoxicity. (a) Normal control group showing normal appearance of glomeruli (G) and tubules (T). (b) Toxic control group exhibiting focal necrosis (N) and lymphatics infilterate (L). (c) 200 mg/kg AMECR group presenting moderate lymphatic infilterate (L). (d) 400 mg/kg AMECR group displaying mild lymphatic infilterate (L). (e) 600 mg/kg AMECR group revealing minute lymphatic infilterate (L).

Likewise, histopathological analysis of kidney sections from control and treated rats was carried out to check the presence of tubular degeneration, tubular interstitial infiltration and tubular necrosis, providing the degree of destruction of tubular cell damage. Normal control group exhibited normal renal parenchyma along with normal tubules and normal glomeruli while, toxic control group exhibited renal parenchyma with focal necrosis and lymphatics infilterate. On contrary to that, renal parenchyma of rats treated with 600 mg/kg of AMECR showed little to no lymphatic infilterate, whereas 400 and 200 mg/kg presented mild to moderate lymphatic infilterate. This reveals highly beneficial effect of AMECR on kidney tissues (Table 5, Figure 3).

**Table 5.** Histopathological analysis of kidneys of gentamicin induced nephrotoxic rats.

Groups	Tubular degeneration	Tubulo-interstitial infiltration	Tubular necrosis
Normal control	Nil	Nil	Nil
Toxic control	Severe	Severe	Moderate
AMECR 200mg/kg	Moderate	Mild	Nil
AMECR 400mg/kg	Mild	Moderate	Nil
AMECR 600mg/kg	Mild	Nil	Nil

### Phytochemical analysis of *Cuscuta reflexa*

Preliminary phytochemical analysis was performed to determine the classes of different phytoconstituents present in AMECR. The results depicted the presence of alkaloids, flavonoids, triterpenoids, saponins and anthraquinones (Table 6).

**Table 6.** Preliminary phytochemical analysis of *Cuscuta reflexa*.

Sr. No.	Name of phytoconstituents	Aqueous methanolic extract
1.	Alkaloids	++
2.	Flavonoids	+++
3.	Triterpenoids	+++
4.	Saponins	+
5.	Anthraquinones	+
6.	Tannins	–

+: Minimum presence of chemical constituents. ++: Normal presence of chemical constituents. +++: Maximum presence of chemical constituents. −: Absence of chemical constituents.

### HPLC analysis of *Cuscuta reflexa*

**Figure 4.**
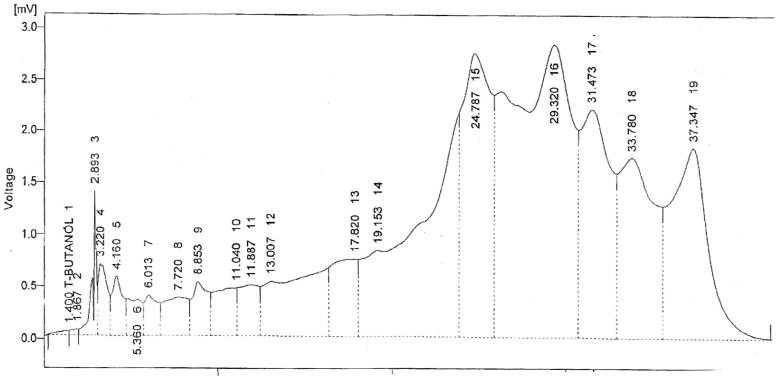
Peaks of phytoconstituents in HPLC analysis.

HPLC analysis was done to determine the presence of phenols and flavonoids. The peaks of all the phytoconstituents were compared with standards. Retention time and quantity were also recorded. The HPLC analysis revealed the presence of *p*-coumaric acid, quercetin, gallic acid, vanillic acid, caffeic acid and trans-4-hydroxy-3-methoxycinnamic acid (Table 7, Figure 4).

**Table 7.** Preliminary HPLC analysis of *Cuscuta reflexa*.

Compound name	Retention time	Area (mV s)	Area (%)	Quantity (Ppm)
Quercetin	2.893	20.776	0.1	1.06
*p*-Coumaric acid	17.280	73.255	2.8	0.94
Vanillic acid	13.007	128.1662	4.9	7.93
Gallic acid	4.160	23.380	0.9	2.82
Caffeic acid	11.887	37.435	1.4	1.78
Trans-4-hydroxy-3-methoxy cinnamic acid	24.787	302.780	11.6	1.05

## Discussion

In the present study, antiarthritic activity of AMECR was evaluated *in vivo* using formaldehyde and turpentine oil-induced arthritis models and *in vitro* using protein denaturation (BSA and egg albumin) methods. Moreover, nephroprotective effect was assessed using gentamicin-induced nephrotoxicity model. Additionally, preliminary phytochemical analysis and HPLC analysis of plant extract was carried out.

Formaldehyde-induced arthritis is the most commonly used model for determining anti-arthritic activity. Formaldehyde causes release of chemical mediators like histamine, serotonin and prostaglandin (Kumar et al. 2008). Inflammation caused by formaldehyde is biphasic in nature, i.e., it shows neurogenic elements which effect brain activity and then lead to tissue-mediated response (Owoyele et al. 2011). Neurogenic phase directly stimulates paw swelling along with centrally mediated effects of pain through the release of pain mediators in the later phase. It has been proclaimed that drugs that works on CNS inhibit both phases uniformly, whilst peripherally acting drugs inhibit late phase (Rao et al. 2005). Inhibition of paw oedema and paw diameter observed in the formaldehyde model may be due to the ability of AMECR to inhibit histamine, serotonin and prostaglandin, which are responsible for inflammation. The maximum dose (600 mg/kg) of AMECR showed marked reduction in joint swelling comparable to that of standard aspirin (100 mg/kg). Moreover, AMECR inhibited both phases of inflammation, therefore it acts centrally.

Turpentine oil-induced arthritis is triphasic in nature. It releases chemical mediator’s, e.g., histamine and serotonin (1st phase), kinin-like substances (2nd phase) and COX and lipoxygenase products (3rd phase) (Kaithwas et al. 2012). The results observed at hourly intervals for 6 h suggest that maximum dose (600 mg/kg) of AMECR manifested noticeable effect in reducing arthritis in rats knee joint, which was comparable with 100 mg/kg aspirin. However, maximum inhibition of oedema was seen during late phase of inflammation, thus suggesting a prominent cyclooxygenase/lipoxygenase inhibitory activity of AMECR (Gupta et al. 1999).

The *in vitro* methods used to check antiarthritic activity included inhibition of protein denaturation using bovine serum albumin and fresh hen’s egg albumin. Previous studies have reported that protein denaturation is one of the leading causes of inflammatory as well as arthritic diseases, which causes production of auto antigens, progressing to certain rheumatic diseases (Chandra et al. 2012). The main mechanism involved in protein denaturation is characterized by changes or alterations in hydrophobic, electrostatic, hydrogen and disulphide bonding among the protein molecules (Grant et al. 1970). Agents that can prevent protein denaturation would be worthwhile for the development of antiarthritic drug. Both AMECR and reference drug showed concentration-dependent inhibition of heat-induced protein (albumin) denaturation, and at higher concentration (800 μg/mL) prominent effects on protein denaturation was produced. From the current findings it could be concluded that AMECR is capable of reducing the production of auto-antigen which indirectly reduces the protein denaturation, and hence alleviate arthritis.

The gentamicin-induced nephrotoxicity implicates renal oxidative stress, accompanied with reduction in renal antioxidant defence mechanisms (Elfarra et al. 1994). The results of this study depict that gentamicin administration to rats produced a typical pattern of nephrotoxicity, manifested by marked increase, while AMECR showed a significant decrease in biochemical parameters. Elevated levels of serum creatinine, urea, uric acid and blood urea nitrogen have been suggested to be an index of renal damage and dysfunction (Finco & Duncan 1976). The curative effect of AMECR on kidney markers can be attributed to its antioxidant property as it has been found that ROS may be involved in the impairment of glomerular filtration rate (Hughes et al. 1996). These results suggest that AMECR has an ability to prevent gentamicin-induced nephrotoxicity in rats.

It was formerly reported that phenolics and flavonoids limit the risk of degenerative diseases associated with oxidative damage (Pandey & Rizvi 2009). In the present investigation, preliminary phytochemical screening determined the presence of alkaloids, flavonoids, triterpenoids, saponins and anthraquinones. Similarly, HPLC analysis revealed the presence of *p*-coumaric acid, quercetin, gallic acid, vanillic acid, caffeic acid and trans-4-hydroxy-3-methoxycinnamic acid. Thus, keeping in view the above discussion, it could be inferred that *Cuscuta reflexa* exerted significant antiarthritic and nephroprotective effects. Yet, the precise mechanism is not known, its protective effect can be linked with the presence of phytoconstituents (phenols, flavonoids). In conclusion, the obtained results justify the traditional use of plant.

## Conclusions

Based on the aforementioned results, it can be concluded that the aqueous methanolic extract of *Cuscuta reflexa* possesses potent antiarthritic and nephroprotective activities that might be owing to the presence of phenols and flavonoids in the plant.
